# Mental Imagery and Food Consumption

**DOI:** 10.3389/fpsyt.2015.00048

**Published:** 2015-04-07

**Authors:** Benjamin Missbach, Arnd Florack, Jürgen König

**Affiliations:** ^1^Department of Nutritional Sciences, University of Vienna, Vienna, Austria; ^2^Department of Psychology, University of Vienna, Vienna, Austria

**Keywords:** mental imagery, habituation, satiation, depletion, mindfulness

## Introduction

One enigmatic capacity of human experience is the ability to travel back and forth in time by using mental simulations. By imagining shapes, forms, and scenes, humans can relive the past and visualize future events (1, 2). Historically, this memory-based mechanism has been discussed in scientific and non-scientific fields. As described by Marcel Proust in his epic work, In Search of Lost Time (3), the sensory experience of gustatory cues, in this case, a sponge-cake called Madeleine, seems to be powerful enough to trigger a cascade of vivid intrusions associated with a particular set of memories (4). Besides anecdotal references, visual mental images are of great interest in the domain of eating behavior because research has shown that (involuntary) mental simulations of intrusions and (voluntary) repetitive mental imagery influence eating behavior. Unraveling the basic mechanisms that underlie mental imagery in the food domain has the potential to provide new insights into the perception and consumption of food.

In this opinion article, we briefly report on the role of mental imagery simulations in eating behavior and its associated pathologies and illustrate how research on mental imaginary has contributed to the current understanding of the cognitive aspects of food intake regulation and satiation processes. Furthermore, we discuss whether guided mental imagery intervention strategies can be integrated into the successful self-regulation of eating behavior and provide a perspective for future research on mental imagery.

## Mental Simulation of Food Consumption and Effects on Judgments and Behavior

Many individuals in modern society live in environments that promote excessive weight gain because of the omnipresence of food (5). The exposure to food automatically prepares individuals for food intake and often evokes thoughts about food, a simulation of food intake, and a strong motivation to consume food (6). Indeed, even the exposure to words related to tempting food can activate a cascade of associations with food (7) and the simulation or reenactment of prior eating occasions (8). Although internal cues of hunger or craving are relevant to the formation of such intrusive thoughts to a large degree, it is clear that thoughts about food consumption are also triggered by consumption-related cues in the environment. Shop design and product presentation in marketing have tapped into these mechanisms by using sensory, textural, and emotional triggers to evoke intrusions, facilitate the simulation of consumption, and increase actual purchases and consumption (9, 10). Recently, research has shown that such marketing practices are not just a creative idea put forth by marketing managers but are supported by concrete evidence for the link between mental simulations and behavior. Elder and Krishna (11) presented participants with a picture of smooth vanilla yogurt in a bowl. They varied whether a spoon was on the right or left side of the bowl. In support of the hypothesis that participants simulate their eating of the yogurt prior to their actual intake in order to forecast the taste of the yogurt, the researchers found that right-handed participants indicated higher purchase intentions when the spoon was on the right side of the bowl compared with when it was on the left side. Hence, the characteristics of the presentation that facilitated the simulation of eating increased the motivation to eat the yogurt. The facilitation effect is not limited to visual perception. For instance, Mitchell and Kahn (12) reported that the ambient odor of a room had a considerable effect on the extent to which individuals thought about the presented products. When the odor was congruent with a product (e.g., chocolate), consumers were more likely to process information about the product. Similarly, Seo and Roidl (13) found that congruent odor enhanced visual attention to odor-congruent food items. In their study, 60 participants were presented with four odors (orange, lavender, coffee, and licorice) prior to and during the presentation of foods via photographic slides. Participants who received olfactory cues looked more frequently and for longer at the corresponding foods than participants in a control condition with no odor presentation. The presentation of food also has an effect on taste assessments. For example, subtle changes of the natural color of an orange juice drink (darker orange hue) decreases liking scores substantially (14). In accordance, manipulating expected brand pronunciation (incongruent brand labeling) was shown to reduce hedonic liking of yoghurts (15). Taken as a whole, the results of the above-mentioned studies correspond with the assumption that conditions that facilitate the simulation of the consumption of attractive food increase interest in the food (e.g., visual attention or processing of information about the food) and the desire for food consumption.

Researchers have recently referred to theories on grounded cognition (16, 17) to explain the effects of perceptions on thoughts and behavior (10). The basic idea of these theories is that the cognitive representation of concepts is grounded in related modal systems. Previous theories on cognition had supposed that cognitions were amodal and, for example, not based on modal representations of motor behavior. These theories had implied that individuals can think about eating in the same way whether or not they can move their mouth and tongue at the current moment. By contrast, the more recent approaches of grounded cognition suppose that thinking about an object is related to perception and motor behavior. This means that humans cannot think about food without simultaneously activating related perceptions and that they simultaneously simulate motor behavior that is related to the stimulus. Hence, the mode of thinking is supposed to be strongly related to the mode of perception and the mode of action. According to the grounded cognition approach, thinking about food or just reading food-related words should evoke mental simulations of eating (7). Recently, such assumptions have received support from neuroscience research. Researchers found that solely the perception of pictures of palatable food led to activations in brain areas associated with gustatory experiences (18) and elevated ghrelin levels (orexigenic hormone) in healthy volunteers (19). Like food cues, ghrelin is one major mediator of food anticipation (20) and recruits the same neuronal circuitry in the dorsomedial and ventromedial hypothalamus (21).

An example of a particularly strong influence of thoughts on behavior is food craving. Food craving is regarded as a strong motivational state that urges individuals to seek and consume a particular kind of food (22), often containing high amounts of sugar or fat (23). When individuals crave food, their thoughts about food are often so intrusive that they have trouble pursuing other goals or focusing on different thoughts. For craving, the causal direction of the influence is bidirectional in the sense that craving has an influence on thought and vice versa. However, it is important to take into account that the mental simulation of food consumption triggered by external cues as described above can result in craving.

## Repeated Mental Simulation and Mindfulness

### Effects of repeated mental simulation on food consumption

An interesting aspect of mental simulations during food perception is that mental simulations do not necessarily lead to increased consumption, but rather, under certain circumstances, they can lead to decreased consumption. Interestingly, repeatedly thinking about food consumption can lead to habituation and a reduced motivation to consume a specific food, just like real consumption (24). Habituation to food is understood as a process that leads to a decrease in both the physiological and behavioral responses to an eating episode and a drop in enjoyment with repeated consumption (24). Several studies (25–27) have found evidence that habituation can take place when individuals repetitively judge food or imagine eating food. For instance, Morewedge et al. (26) asked participants to think either 3 or 30 times about eating M&M’s. Later, participants were allowed to eat M&M’s. Participants who thought about eating the candy 30 times ate less of the product than those who thought about it 3 times and those in a control condition (who imagined throwing coins into a laundry machine). Like other habituation effects, this effect was shown to be sensory-specific and was only present when the imagined food was congruent with the consumed food. The effect did not show when the imagined food (e.g., M&M’s) was not the same as the consumed food (e.g., cheese cubes) [(26); Experiment 4]. Similar sensory-specific characteristics between imagined and consumed foods are therefore fundamental for food habituation to occur. In our recent study, we found that habituation after imagined eating needs mental resources as habituation during actual eating does (28). Several studies have indicated that habituation is based on a memory process that needs cognitive capacity to occur. Hence, when individuals are distracted during eating, for example, when they watch TV during eating (29, 30), they habituate less to food and eat more. We found that a depleting task had a similar effect on habituation after imagined food consumption. In our study, we used a mathematical counting task (31) to deplete participants prior to simulating the eating of 18 walnuts in one condition. We showed that participants in this depletion condition did not habituate to the mental simulation, and habituation was blocked, whereas they habituated in a condition without depletion.

### Effects of mindfulness on food choice

As reported above, repeatedly imagining food consumption can reduce the desire to consume the food (26). But repetitive thoughts about food consumption are not the only way to reduce the desire to consume a particular kind of food. Also important is *how* individuals think about the food. While a single vivid image of the consumption increases the influence of impulsive responses on food choice, the influence of impulsive responses on food choice decreases when consumers think about the reasons for their food choice (32). Similarly, mindful attention to thoughts about food consumption can reduce choices for unhealthy food items that are impulsively preferred by consumers. For example, Papies and colleagues (6) instructed participants to regard their thoughts from a metacognitive perspective as temporary constructions that appear and disappear. When this mindful attention was applied to food consumption, it reduced impulsive tendencies to approach food (6) and also promoted choices of healthy food (33).

### Repeated mental simulation and mindfulness as interventions

It is important to note that the effects of mindfulness are conceptually different from effects of repeated mental simulation. First, repeated mental simulation, but not mindfulness, needs repetition to show effects. Second, repeated mental simulation shows specific effects on a particular kind of food (26) but this is not the case for mindful attention (6). At present, it can be assumed that the habituation effects of repeated mental simulation reflect a memory process that leads to inhibition after some length of imagining exposure to food, whereas mindfulness might be more likely to block the tempting simulation of food consumption. Both processes are of interest for practice. Mindfulness could be used as a method for reducing unhealthy food intake. By contrast, repeatedly imagining food consumption might reduce food intake when consumption has already begun. For example, to increase habituation to a particular kind of food, individuals could think about consuming an unhealthy food that they usually prefer to eat (e.g., chocolate) repeatedly across consecutive days. However, while such long-term effects were demonstrated with exposure to real food (34), it is still a task for future research to study the long-term effects of imagined food consumption. But there is no doubt that the relevance of habituation processes is obvious if we consider recent research that has shown that overweight children habituate more slowly during consumption than non-overweight children (35). Against this background, research on repeated mental simulations could provide a promising starting point from which to advance interventions in food consumption.

## Conclusion

Current research on food intake behavior regulation is driven by a vital need to understand the successful and unsuccessful self-regulation of food consumption. Integrating the cognitive mechanisms of mental imagery and mindfulness that guide eating behaviors may be one important milestone for enhancing current models of food intake regulation. As proposed by Redden (36), a general model should include reflective (memory recall inferences, metacognitions) and perceptual (adaptation, habituation) components of satiation. He argues that satiation is partially constructed in the moment on the basis of external cues that interact with each other in a specific eating situation. Research on mental imagery and mindfulness can help us understand eating behavior and to design individual-level interventions. It can also help us understand why some individuals are more successful self-regulators than others (37).

## Conflict of Interest Statement

The authors declare that the research was conducted in the absence of any commercial or financial relationships that could be construed as a potential conflict of interest.
